# A graph-based approach to construct target-focused libraries for virtual screening

**DOI:** 10.1186/s13321-016-0126-6

**Published:** 2016-03-15

**Authors:** Misagh Naderi, Chris Alvin, Yun Ding, Supratik Mukhopadhyay, Michal Brylinski

**Affiliations:** Department of Biological Sciences, Louisiana State University, Baton Rouge, LA USA; Department of Computer Science and Information Systems, Bradley University, Peoria, IL USA; Department of Physics and Astronomy, Louisiana State University, Baton Rouge, LA USA; Department of Computer Science and Engineering, Louisiana State University, Baton Rouge, LA USA; Center for Computation and Technology, Louisiana State University, Baton Rouge, LA USA

**Keywords:** Molecular synthesis, Virtual screening, Target-focused libraries, *e*Synth, Chemical space, Targeted drug discovery

## Abstract

**Background:**

Due to exorbitant costs of high-throughput screening, many drug discovery projects commonly employ inexpensive virtual screening to support experimental efforts. However, the vast majority of compounds in widely used screening libraries, such as the ZINC database, will have a very low probability to exhibit the desired bioactivity for a given protein. Although combinatorial chemistry methods can be used to augment existing compound libraries with novel drug-like compounds, the broad chemical space is often too large to be explored. Consequently, the trend in library design has shifted to produce screening collections specifically tailored to modulate the function of a particular target or a protein family.

**Methods:**

Assuming that organic compounds are composed of sets of rigid fragments connected by flexible linkers, a molecule can be decomposed into its building blocks tracking their atomic connectivity. On this account, we developed *e*Synth, an exhaustive graph-based search algorithm to computationally synthesize new compounds by reconnecting these building blocks following their connectivity patterns.

**Results:**

We conducted a series of benchmarking calculations against the Directory of Useful Decoys, Enhanced database. First, in a self-benchmarking test, the correctness of the algorithm is validated with the objective to recover a molecule from its building blocks. Encouragingly, *e*Synth can efficiently rebuild more than 80 % of active molecules from their fragment components. Next, the capability to discover novel scaffolds is assessed in a cross-benchmarking test, where *e*Synth successfully reconstructed 40 % of the target molecules using fragments extracted from chemically distinct compounds. Despite an enormous chemical space to be explored, *e*Synth is computationally efficient; half of the molecules are rebuilt in less than a second, whereas 90 % take only about a minute to be generated.

**Conclusions:**

*e*Synth can successfully reconstruct chemically feasible molecules from molecular fragments. Furthermore, in a procedure mimicking the real application, where one expects to discover novel compounds based on a small set of already developed bioactives, *e*Synth is capable of generating diverse collections of molecules with the desired activity profiles. Thus, we are very optimistic that our effort will contribute to targeted drug discovery. *e*Synth is freely available to the academic community at www.brylinski.org/content/molecular-synthesis.

**Graphical abstract:**
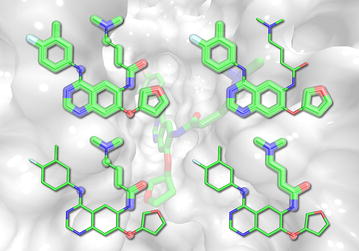
Assuming that organic compounds are composed of sets of rigid fragments connected by flexible linkers, a molecule can be decomposed into its building blocks tracking their atomic connectivity. Here, we developed eSynth, an automated method to synthesize new compounds by reconnecting these building blocks following the connectivity patterns via an exhaustive graph-based search algorithm. *e*Synth opens up a possibility to rapidly construct virtual screening libraries for targeted drug discovery

## Background

Due to extreme costs of high-throughput screening, many drug discovery projects commonly employ inexpensive computations to support experimental efforts. In particular, virtual screening, a technique that shows great promise for lead discovery, has become an integral part of modern drug design pipelines [1, 2]. Here, the idea is to considerably reduce the number of candidate compounds that need to be tested experimentally against a protein target of interest. Due to advances in computer technology resulting in constantly increasing computational power, virtual libraries comprising many thousands of compounds can be rapidly evaluated in silico prior to experimental screens and at a fraction of the cost. Virtual screening approaches, historically divided into ligand- and structure-based algorithms, prioritize drug candidates by estimating the probability of binding to the target receptor [3]. Among many methods developed to date, docking-based techniques are valuable tools for lead identification [4]. These algorithms rank compounds by modeling the binding pose of a query molecule in the binding pocket of the target protein, followed by the prediction of binding affinity from molecular interactions. There are many examples of successful applications of virtual screening to develop compounds with desired bioactivities [5, 6].

Computer-aided drug discovery traditionally utilizes large compound libraries. For example, the ZINC database is one of the most comprehensive repositories of commercially available compounds for virtual screening [7]. It currently features over 35 million compounds in ready-to-dock formats. These large generic collections of low molecular weight organic compounds provide a sufficient diversity to perform virtual screening against any molecular target, however, the vast majority of compounds will have a very low probability to exhibit the desired bioactivity for a specific target protein. Furthermore, considering the imperfections of compound ranking by virtual screening algorithms [8], the top-ranked subset of compound library may contain a very few active molecules. Consequently, the chances to identify novel, high-quality leads from large compound repositories are low. For instance, an internal analysis of the Abbott compound collection suggested that <4 % of the compounds in their library have the potential to yield novel kinase hinge-binders [9]. To address these issues, there has been a significant effort to augment existing collections with those compounds having a higher potential to bind to a specific target of interest. On that account, the trend in library design has shifted to include a strong focus on the target class in addition to diversity and drug-likeness criteria [10].

A target-focused library is a screening collection of compounds specifically tailored to modulate the function of a particular target or a protein family [11, 12]. There are a variety of approaches developed to date to design target-specific focused libraries against, e.g. protein kinases, ion channels, G-protein coupled receptors (GPCRs), nuclear receptors, and protein–protein interfaces. These libraries not only reduce waste by eliminating compounds that are unlikely to bind to target proteins, but often lead to the increased potency and specificity of binders, as demonstrated for c-Src kinase [13]. Several approaches employ molecular docking to determine target-specific thresholds that can be used as filters in virtual screening. This strategy was experimentally validated on the kinase-targeted library of 1440 compounds and 41 kinases from five different families, demonstrating a 6.7-fold higher overall hit enrichment compared to a generic compound collection [14]. Furthermore, a structure-based modeling was used to create a small, focused library against *Chlamydophila pneumoniae*, a common pathogen recently linked to atherosclerosis and the risk of myocardial infarction [15]. Encouragingly, the experimentally determined hit rate for the targeted library was 24.2 %, which is considerably higher than that expected for a generic library. Similar to structure-based approaches, ligand-based techniques can also be used in the focused library design, as shown for the GPCR family [10]. Compared to large and diverse screening libraries, using relatively small and targeted collections significantly improves the odds of finding potential drug candidates, thus further reduces the costs of drug discovery.

Target-focused libraries are either designed or assembled upon some understanding of a specific protein target or a protein family. These collections are often compiled from larger, more diverse libraries using either molecular docking (structure-based approach) or ligand fingerprint similarity (ligand-based approach). The former employs structural, sequence and mutagenesis data, whereas the latter is based on the biomolecular properties derived from known ligands, offering a useful way of “scaffold hopping” from one ligand class to another [16]. Target-focused libraries are often constructed around a single scaffold with one or more positions used to attach various chemical moieties or side chains. Although this approach can result in millions of different compounds [17], the chemical space remains largely unexplored, therefore, truly novel compounds will not be discovered. On the other hand, combinatorial chemistry methods can produce a vast collection of divers compounds, so vast that only a tiny fraction of them could be explored, even using supercomputers. One can hardly imagine screening the chemical universe containing from 10^12^ to 10^180^ drug-like compounds [18]. Therefore, techniques to design chemical libraries covering pharmacologically relevant regions are needed [19]. These methods hold a promise to advance our knowledge of biological processes leading to new strategies to treat diseases.

Compiling focused libraries by molecular synthesis is essentially a combinatorial problem that can be addressed using graph theory. These techniques have been already extensively used in computer science and artificial intelligence for the synthesis of plans [20], problems and solutions in geometry [21], hardware from specifications [22], and communication protocols [23]. Graph-based approaches also have a wide range of applications in drug discovery including the analysis of chemical structures to better understand the common features of drug molecules [24], the design of novel bioactive compounds with the desired pharmacological profiles [25], the structure-based modeling of protein flexibility upon ligand binding [26], the investigation of systems-level drug-target interaction networks [27], and drug repositioning [28].

In this study, we propose a new method to computationally synthesize molecules for virtual libraries, called *e*Synth. In essence, an exhaustive graph-based search algorithm is used to reconnect chemical building blocks procured from bioactive compounds following realistic connectivity patterns. Rather than focusing on a certain scaffold, the moieties used for synthesis come from active ligands of a specific target protein. Thus, the resulting chemical space is highly diverse, yet targeted. Given a set of initial molecules, *e*Synth generates new compounds to populate the pharmacologically relevant space. In order to evaluate the performance of *e*Synth, we conducted a series of benchmarking calculations against the Directory of Useful Decoys, Enhanced (DUD-E) dataset. First, in a self-benchmarking test, we validate the correctness of the search algorithm with the objective to recover a molecule from its building blocks. Further, the capability to discover novel scaffolds is assessed in a cross-benchmarking test. Here, bioactive compounds for each DUD-E target are first clustered into chemically dissimilar groups. Subsequently, each group considered as the validation set is reconstructed using dissimilar molecules pooled from other clusters. This protocol mimics a real application, where one expects to discover novel compounds based on a small set of already developed bioactives. Equally important, *e*Synth allows adding active subunits to an existing compound in order to generate a large library of prototypes of the modified ligand. Such libraries can be examined by molecular docking to explore those modifications yielding the highest binding affinity to the protein target.

## Implementation

### Molecular fragments

**Fig. 1:**
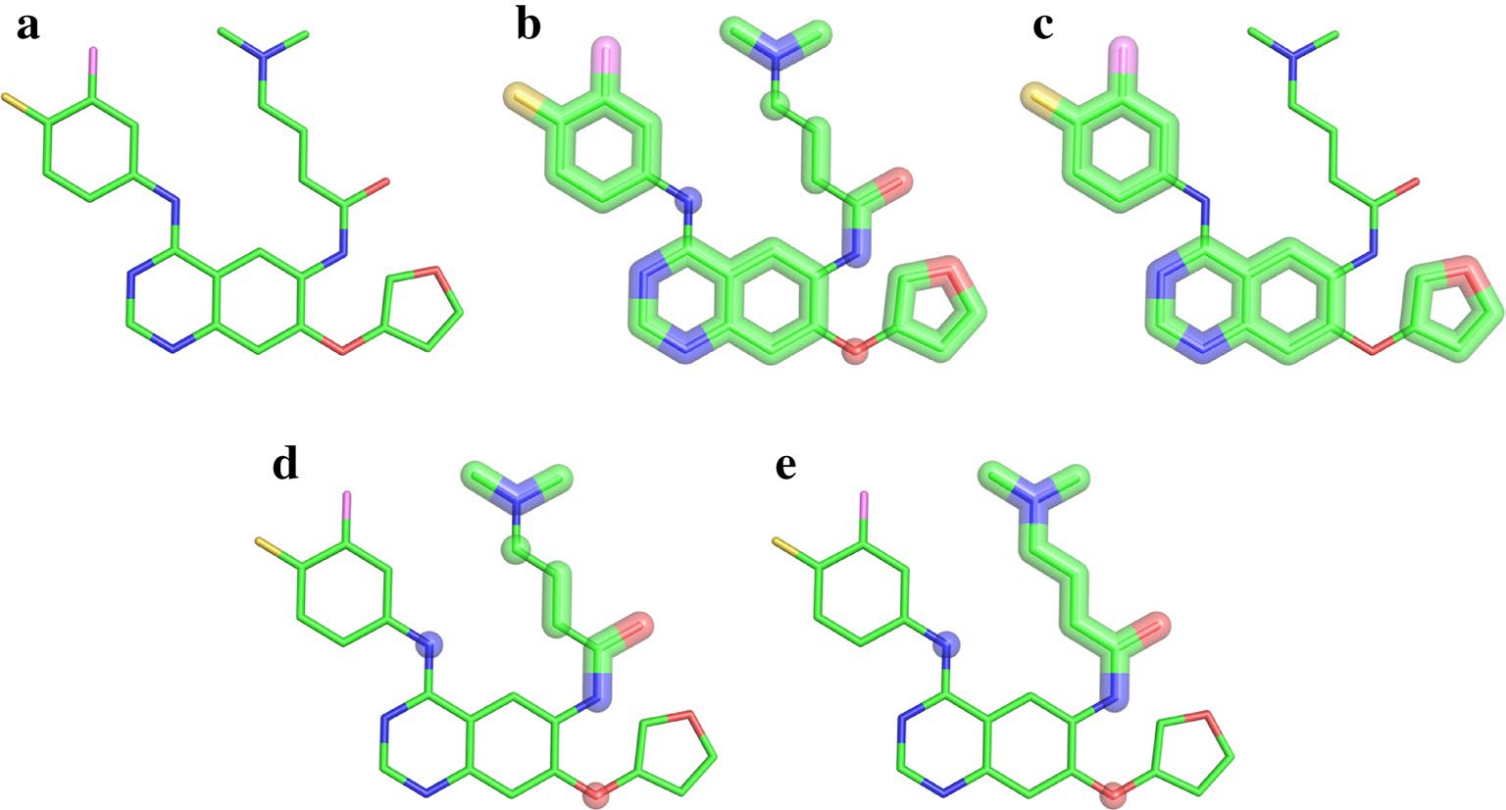
Decomposing organic compounds into molecular fragments. Assuming that organic compounds are composed of sets of rigid fragments connected by flexible linkers, a molecule can be decomposed into its building blocks tracking the atomic connectivity. **a** A stick representation of afatinib. Extracting rigid fragments: **b** all rigid fragments are shown as *thick lines*, **c** only those rigid fragments composed of four or more atoms are retained. Extracting flexible linkers: **d** small fragments connected by rotatable bonds, **e** small linkers are merged to form longer fragments, a single atom can act as a linker as well. The following colors are used for atom types: carbon—*green*, nitrogen—*blue*, oxygen—*red*, fluorine—*yellow*, and chlorine—*pink*

We developed a procedure for the automatic identification and extraction of molecular fragments from chemical compounds. An example decomposition procedure is shown in Fig. 1. The extraction process utilizes the PDBQT file format containing a central rigid fragment, labeled as ROOT, from which zero or more rotatable bonds protrude. The sets of atoms connected through rotatable bonds are organized as BRANCHes, and at the beginning and end of each BRANCH section, the serial numbers of the two atoms forming a rotatable bond are recorded. First, we identify all rigid moieties (Fig. 1b), where a rigid fragment is defined as a set of at least four non-hydrogen atoms connected by non-rotatable bonds (Fig. 1c). The remaining parts are extracted as flexible linkers (Fig. 1d). If two linker fragments are attached to each other, these will be connected to form a longer linker (Fig. 1e). Failing to construct longer linkers from shorter fragments would limit the library to contain only very short linkers. Furthermore, we track the connectivity between individual fragments, so that chemically feasible compounds can be synthesized using a graph-based algorithm. Every fragment is stored in the Structure Data Format (SDF) containing the 3D coordinates of all atoms and the corresponding atomic types as well as the connectivity information. The following SYBYL chemical types [29] are used for ligand atoms: carbon (*C*.1, *C*.2, *C*.3, *C*.ar and *C*.cat), nitrogen (*N*.1, *N*.2, *N*.3, *N*.4, *N*.am, *N*.ar and *N*.pl3), oxygen (*O*.2, *O*.3 and *O*.co2), phosphorous (*P*.3), sulfur (*S*.2, *S*.3, *S*.O and *S*.O2), and halogens (*Br*, *Cl*, *F*, *I*).

### Connectivity information

**Fig. 2:**
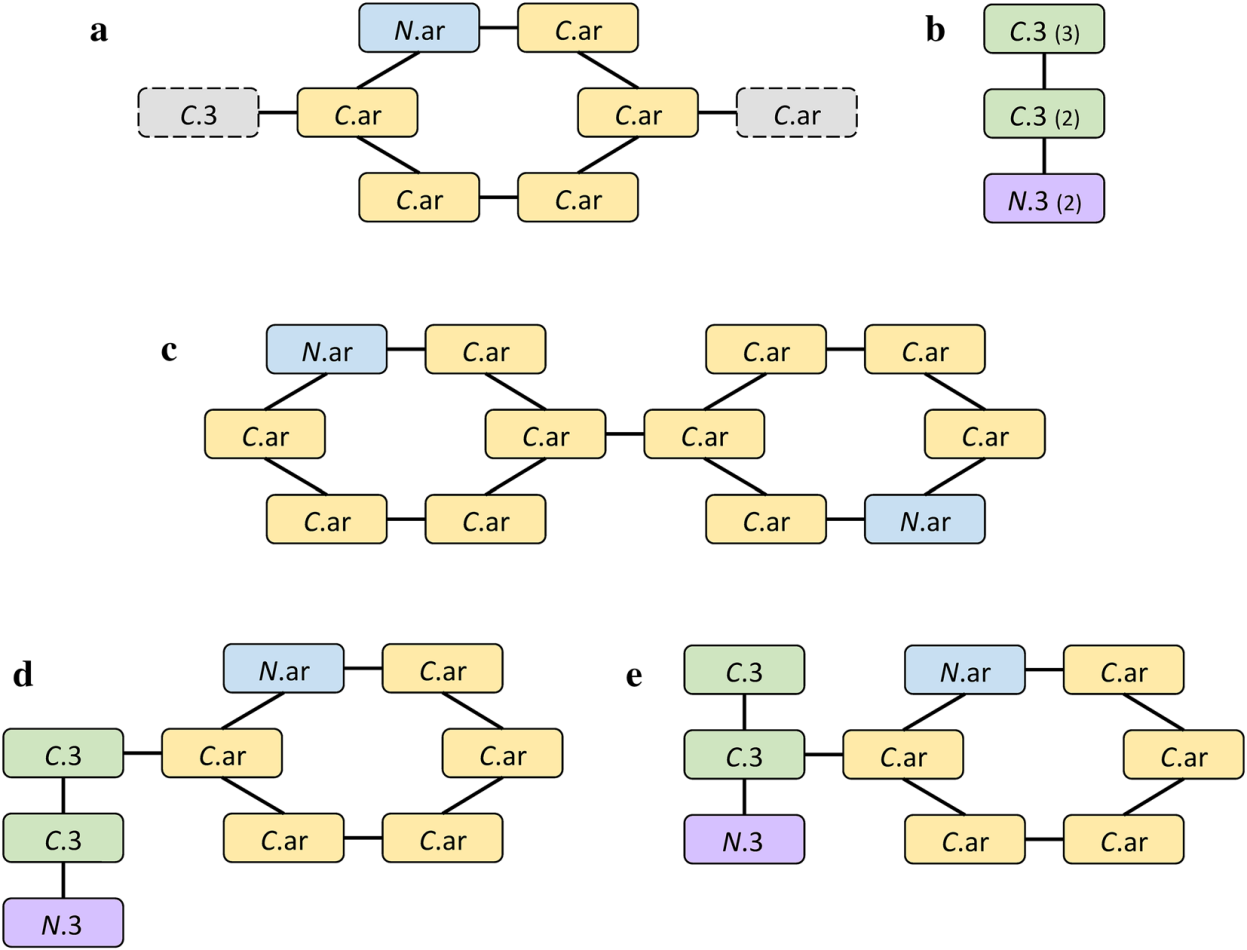
Graph representation of rigids and linkers. Sample molecular fragments: **a** a rigid fragment, pyridine, with six constituent atoms in the *bold outline* and two possible connections to *C*.3 and *C*.ar in the *dashed outline*, **b** a three-atom linking fragment containing *C*.3 carbon with up to 3 connections, *C*.3 carbon with up to 2 connections, and *N*.3 nitrogen with up to 2 connections. Examples of *2*-molecules: **c** two identical rigids connected to each other, **d**, **e** two possible ways of connecting rigid and linker fragments shown in **a**, **b**

Figure 2 illustrates the graph representation of rigids and linkers. A rigid fragment carries connectivity information indicating those atoms from which a rigid fragment was originally branched and the corresponding atom types it was connected to (Fig. 2a). On the other hand, linkers contain information only on the number of allowed contacts at every atom, which is sufficient to create bonds with rigid fragments (linkers cannot bind to each other). The number of connections in a linker cannot exceed the maximum number of covalent bonds. Thus, we saturate a linker with hydrogen atoms and report the maximum number of bonds allowed for each atom in the linker file, e.g. *N*.3 atom shown in Fig. 2b can bind at most two atoms that belong to rigid fragments accepting *N*.3. Noticeably, long linkers with the extensive connectivity pose a risk of expanding the molecular search space to an unmanageable size. Therefore, unsaturated linkers can also be built to store only the number of original connections, regardless of the maximum capacity of their atoms to create covalent bonds. In contrast to saturated linkers, using unsaturated linkers with substantially less connectivity considerably restricts the search space.

### Fragment consolidation and pruning

Redundancy is removed from molecular fragments extracted from multiple compounds by consolidating the connectivity information and deleting identical moieties. For instance, if different parent molecules have a similar fragment, it suffices to have only one file for this moiety. However, if this fragment is connected to different atom types in distinct parent molecules, all possibilities should be retained. Therefore, this information is deposited as one copy of the fragment representing all possible connections. This is shown in Fig. 2a, where the aromatic ring is the rigid fragment (solid round boxes) that was connected to *C*.3 in one parent molecule and to *C*.ar in another (dashed round boxes); this information is consolidated to create only one rigid moiety with two possible connections.

### Exhaustive molecular synthesis with *e*Synth

*e*Synth considers molecular bonding over a given set of rigid and linker fragments restricted by the laws of chemistry. Molecular synthesis is a fixed-point approach to generate a complete set of molecules given a set of fragments. The component algorithms of *e*Synth are described in the following sections.

A fragment-based approach to synthesis can result in an infinite molecular search space unless an upper bound for molecular size is specified. Even with reasonable upper bounds imposed on the molecule size, the synthesis process may result in 10^8^ molecules or more. It is therefore highly desirable to develop an efficient algorithm for molecular synthesis that is complete, i.e. all possible molecules that can be synthesized under chemical and physical constraints are guaranteed to be generated. For expository purposes, we will refer to a *k*-molecule as a molecule that is composed of *k* molecular fragments. Algorithm 1 uses a level-based approach to molecular synthesis, where all molecules in a level are composed of the same number of fragments.
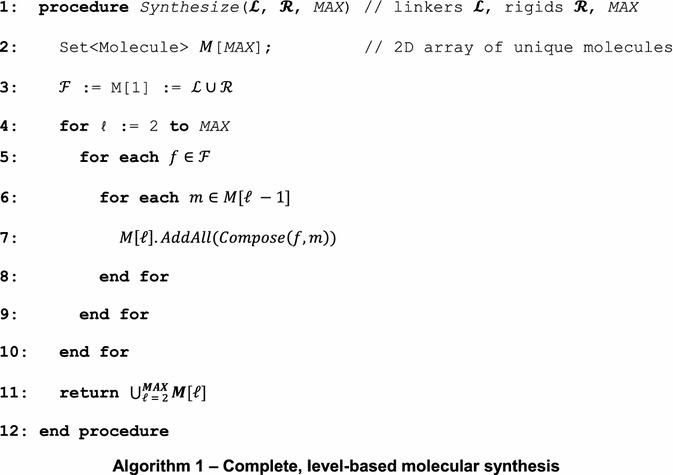


In Algorithm 1, line 3 initializes the synthesis process by storing *1*-molecules (i.e. fragments) in the array *M* (at index 1). On lines 4 to 10, we exhaustively synthesize each new level from *2*- to *MAX*-molecules, where *MAX* is an upper bound parameter set by the user. For simplicity, we store all *k*-molecules at index *k* in *M*. The synthesis process is performed by the $Compose\left( {m_{1} , m_{2} } \right)$ function which takes two molecules *m*_1_ and *m*_2_ and combines them together in all possible orientations as dictated by allowable bonding vertices (connectivity information) in the graph representation of each molecule. See Fig. 2c–e for examples of molecular bonding with the *Compose* function. *Compose* returns a set of molecules that meet the stated constraints, including Lipinski [30] compliance, to be added to the appropriate set of *k*-molecules. Last, on line 11, we combine the sets of all synthesized molecules into a single collection that is returned.

The level-based approach to molecular synthesis described in Algorithm 1 is malleable depending on computational constraints. For example, Algorithm 1 implies that the synthesis of level *k* must complete prior to level *k* + 1 starting. However, an astute observer will recognize that Algorithm 1 can easily be modified for a multi-threaded approach in which level *k* is a producer for level *k* + 1, the consumer. Thus, if each level maintains a thread acting as producer and consumer, the synthesis process can be expedited.

Similarly, we may introduce a bounded alternative of Algorithm 1. In Algorithm 2 (the bounded alternative), we maintain an array of worklists (line 3), one for each level that has an explicit capacity. If we reach the capacity of a worklist at level ℓ, we forgo processing the remaining items at level ℓ and inductively complete processing of all molecules at level ℓ + 1 (Line 12). Otherwise, from lines 15 to 17 we compose a molecule from level ℓ with all of the fragments in ${\mathbf{\mathcal{F}}}$ into level ℓ + 1 molecules as before. We note that the approach in Algorithm 2 is appropriate for either serial or parallel syntheses depending on the availability of computational resources.
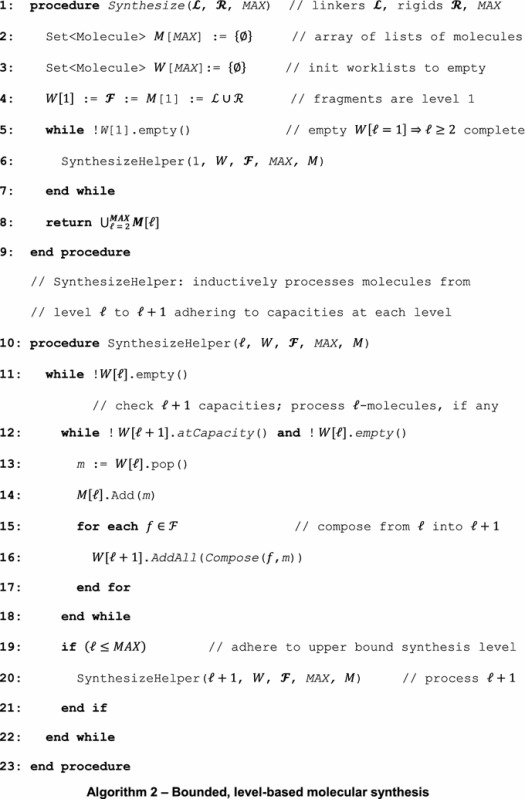


### Molecular filtration with Bloom filters

Molecular synthesis is limited by physical restrictions on molecules, but more so time and space. An efficient synthesis must overcome time and space considerations, generate molecules within the physical restrictions, but do so without redundancy. Using either Algorithm 1 or Algorithm 2 results in a significant redundancy present in synthesized molecules. A typical synthesis scenario from a basis of fragments will generate hundreds of millions of molecules, which makes storing these molecules in memory infeasible. Therefore, eliminating molecular redundancy requires a memoryless technique; the synthesis requires a series of Bloom filters [31].

A Bloom filter is a probabilistic data structure that is efficient in terms of time and space. Although Bloom filters are well-studied, we describe their use in our synthesis domain. The main purpose of a Bloom filter is to determine whether an element is in a given set. Let ${\mathcal{M}}$ be a set of molecules and *M* a molecule. A Bloom filter is guaranteed to answer the query $M \in {\mathcal{M}}$ if molecule *M* is an element in set ${\mathcal{M}}$. Since a Bloom filter is a probabilistic data structure, it is subject to false positives, i.e. a query may return $M \in {\mathcal{M}}$ when in fact $M \notin {\mathcal{M}}$, however, the rate of false positives can be controlled. Although we omit some details of a Bloom filter, we consider the rate of false positives. A Bloom filter is based on the number of bits in the filter array *b*, the number of distinct hash functions *h*, and the number of elements *n* we expect to insert into the filter. Assuming all hash functions hash elements uniformly to all *b* bits in the target array, the rate of false positives for an element *M* not in a set ${\mathcal{M}}$ is given by $P\left( {M \notin {\mathcal{M}}} \right) = \left(1 - e^{-nh/b}\right)^{h}$. It can be shown that to minimize the rate of false positives, the required number of hash functions *h* is given by $h = \frac{b \times \ln 2}{n}$. If *p* is the desired false positive rate, it can also be shown that the required number of bits is $b = - \frac{n \times \ln p}{{\left( {\ln 2} \right)^{2} }}$ [31]. Consider a molecular Bloom filter *F* in which we tolerate a 1 % false positive rate for 10^8^ molecules. In this case, we require $b = 9.585 \times 10^{8} \approx 120$ megabytes with *h* = 7 hash functions. This means each addition of a molecule to *F* and each query on *F* is subject to the worst case of *O*(*h*) = 7 hashings.

Molecular synthesis requires a string representation of molecules. In particular, a molecule *M* is represented using the Simplified Molecular-Input Line-Entry System (SMILES) specification [32] in the Bloom filter. We can modify the *Compose* function in Algorithm 2 by including several Bloom filters, a single, overall filter *F* and a filter *F*_ℓ_ for each level. When a level ℓ molecule *M* is synthesized, we first check whether it has been previously synthesized by querying *F*_ℓ_. If the molecule has not been synthesized (*M* ∉ *F*_ℓ_), we add *M* to *F*_ℓ_ and query *F*. If *M* ∉ *F*, we add *M* to *F* and proceed as in Algorithm 2 by adding *M* to the level-ℓ queue to be processed into level-(ℓ + 1) molecules. Clearly, the global Bloom filter *F* requires the most memory, but ensures that molecules containing different number of fragments with the same SMILES representation are filtered as redundant.

### Implementation of *e*Synth

**Fig. 3:**
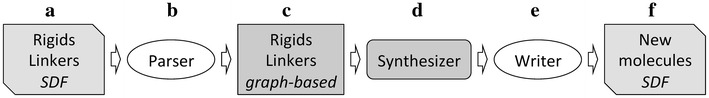
Implementation of *e*Synth. Input rigid and linker fragments in SDF format (**a**) are parsed (**b**) into the graph-based representation (**c**). Synthesizer (**d**) is the main engine to generate new molecules, which are subsequently passed to the Writer (**e**) component and output in SDF format (**f**)

The architecture of *e*Synth shown in Fig. 3 reflects a simple input/output paradigm with a black-box synthesizer. The input to *e*Synth is a set of rigid and linker fragments in SDF format (Fig. 3a). Each SDF file is parsed (Fig. 3b) using some functionality of Open Babel into a graph-based representation of the corresponding rigids and linkers (Fig. 3c). From the set of linkers and rigids, the Synthesizer (Fig. 3d) implements Algorithm 2 to construct new compounds (Fig. 3f). Each synthesized molecule is output using Open Babel in the Writer component (Fig. 3e).

One significant challenge in developing *e*Synth was integrating Open Babel to handle molecules. It is clear from Open Babel documentation and our own experience that even a simple operation such as molecular addition is thread-unsafe. It is necessary, therefore, to treat Open Babel as a singleton resource; we use Open Babel in a limited capacity to handle input and output. We then employ a local representation of compounds and fragments (optimizing memory with bit fields) to ensure that the Synthesizer is independent of Open Babel. Open Babel creates an undesirable bottleneck in the multi-threaded implementation of *e*Synth and thus a serial execution is superior.

### Validation datasets and procedures

In order to evaluate the performance of *e*Synth, a series of benchmarking calculations were conducted against the DUD-E dataset [33]. Here, we use 20,408 bioactive compounds for 101 receptor proteins representing many important drug targets. First, we validated the correctness of the search algorithm using a self-benchmarking test. Subsequently, we performed a cross-validation test to evaluate the capability of *e*Synth to generate bioactive molecules with novel chemical structures.

In the self-benchmarking test, each active compound in the DUD-E library was decomposed into fragments and the molecular synthesis was performed. Parent compounds are compared to those constructed by *e*Synth using molecular fingerprint matching with the chemical similarity assessed by the Tanimoto coefficient (TC) [34, 35]. The cross-validation test mimics a real application, where novel compounds are expected to be discovered based on a small set of known bioactive molecules. Here, bioactive compounds for each DUD-E target were first clustered into a collection of chemically dissimilar groups using SUBSET [36] and a TC similarity threshold of 0.7. Subsequently, each cluster was selected as a validation set and molecular fragments from the remaining clusters were used by *e*Synth to build new molecules. The performance of *e*Synth is evaluated using the fraction of successfully reconstructed validation compounds using fragments extracted from chemically different molecules. Due to the large size of compound datasets generated by *e*Synth, we first used Open Babel [37] to filter out those molecules that are dissimilar to the validation compounds with TC < 0.5. Next, 3D atomic coordinates were generated for the synthesized molecules using obgen from the Open Babel package. A build-up algorithm to find atomic correspondence between chemical structures that calculates 2D-TC based on the identified the maximum common substructure (kcombu) [38] was then applied to measure the topological similarity between the filtered subset of synthesized molecules and the validation compounds.

## Results

### Search algorithm and the computational efficiency

*e*Synth generates target-focused libraries directly form ligands known to bind to a particular target protein or a family of proteins. The synthesis protocol first decomposes bioactive compounds into the non-redundant sets of chemical building blocks and then exhaustively combines these fragments to generate new molecules. We define two types of fragments, rigid moieties and flexible linkers; each unique fragment is accompanied by the connectivity information. In case of identical fragments extracted from different molecules, the connectivity information is consolidated to produce a complete list of possible connections for every atom in this fragment. As a result, the fragment library compiled for a given protein target is non-redundant and representative.

Each fragment is converted to a graph-based representation of a chemical entity, where nodes correspond to atoms and undirected edges represent chemical bonds. Nodes in the molecular graph are annotated with the connectivity information. For instance, a rigid fragment shown in Fig. 2a can produce *para*-substitutions with two moieties attached through their *C*.3 and *C*.ar atoms at opposite positions on the heterocyclic aromatic ring, whereas Fig. 2b shows a 3-atom linker that can form up to 7 bonds with rigid fragments (3 on top *C*.3 + 2 on middle *C*.3 + 2 on bottom *N*.3). From a set of unique fragments represented as graphs with annotated nodes, the synthesis algorithm constructs increasingly larger molecules, such as those presented in Fig. 2c–e.

**Fig. 4:**
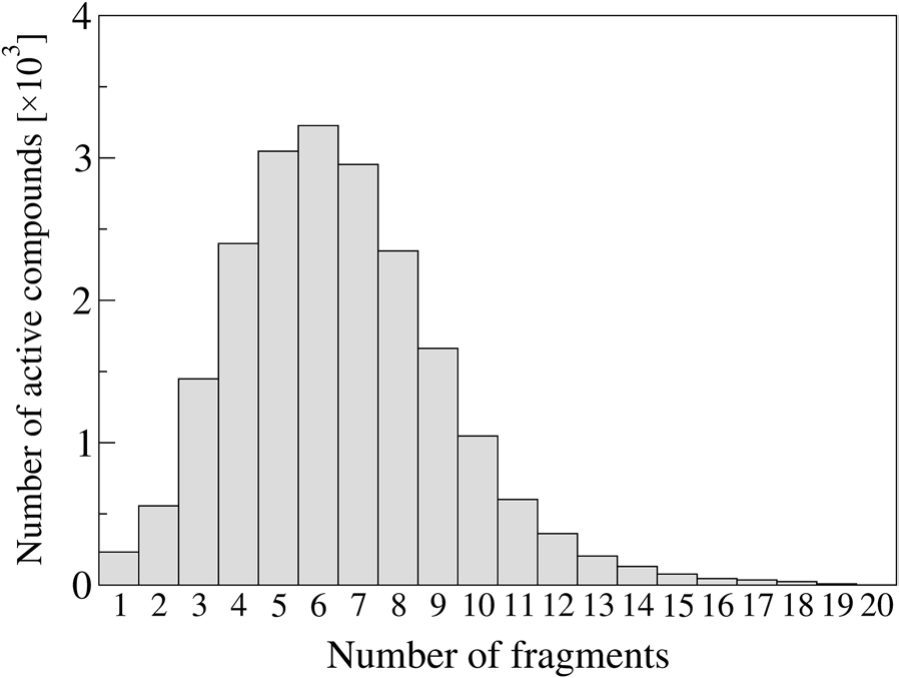
Number of fragments in DUD-E compounds. Histogram of the number of fragments in active compounds from the DUD-E dataset

Our initial implementation of the molecular synthesis described in Algorithm 1 is non-optimal due to considerable limitations on the wall time and memory space. For example, implementing a strict, either serial or parallel level-based approach results in ever-increasing memory requirements. Experimentally, we encounter this sharp increase of the number of generated molecules around levels 9 through 11, which can be expected considering the mode in the distribution of molecular fragments in the DUD-E database presented in Fig. 4. Due to the exponential growth and the width of the chemical space, a solution was required to overcome the strict level-based approach.

On that account, we optimized the molecular synthesis by (1) implementing the bounded, level-based algorithm described in Algorithm 2, (2) reducing redundancy with Bloom filters, (3) restricting the connectivity using unsaturated linkers to narrow the width of the search space in order to gain depth, and (4) eliminating compounds violating the Rule-of-Five. The bounded, level-based synthesis algorithm imposes an explicit capacity on each level, so that all levels can be explored; it also ensures the termination of the synthesis procedure. Bloom filters provide computationally efficient mechanisms to eliminate those molecules that have already been synthesized. Furthermore, long, saturated linkers pose a considerable risk of expanding the molecular search space to an unmanageable size, therefore, we introduced unsaturated linkers accepting only those connections that were originally present in their parent molecules. In contrast to saturated linkers that can form all chemically possible bonds with rigid fragments, unsaturated linkers significantly restrict the search space, dramatically improving the computational efficiency. Finally, using the Rule-of-Five ensures that the synthesized compounds have drug-like properties. However, in order to test the drug likeliness of a molecule prior to its synthesis, Lipinski’s descriptors need to be estimated from molecular fragments, which is discussed in the following section.

### Prediction of physicochemical properties

According to Lipinski’s Rule-of-Five, a drug candidate should have a molecular mass (MW) <500 Da, no more than 10 hydrogen bond acceptors (HBA), no more than 5 hydrogen bond donors (HBD), and the octanol–water partition coefficient (logP) below 5 [30]. It is important to note that nearly half of active compounds in the DUD-E dataset (9332 out of 20,402) would not pass this filter with the default cutoffs. To mitigate this problem, we modified thresholds for MW and logP to ensure that the majority of actives comply with the drug-likeness criteria. Specifically, using MW ≤ 570, HBD ≤ 5, HBA ≤ 10 and logP ≤ 7.2 increases the number of compliant molecules to 18,104 (88.7 % of DUD-E actives). We refer to these values as a modified Rule-of-Five.

Decomposing the library of 20,408 bioactive DUD-E compounds resulted in 67,801 linkers and 65,507 rigid fragments. Due to the number of possible combinations growing exponentially with the number of molecular fragments, a modified Rule-of-Five is applied to exclude those compounds that do not satisfy drug-likeness criteria. It is therefore critical to rapidly estimate these properties directly from molecular fragments used to construct chemical compounds in order to prevent the synthesis of non-compliant molecules. This approach restricts the molecular search only to those compounds having drug-like properties. The additive nature of Lipinski’s descriptors allows for MW, HBD, HBA and logP of the synthesized molecules to be estimated directly from values pre-calculated for the fragment library.

**Fig. 5:**
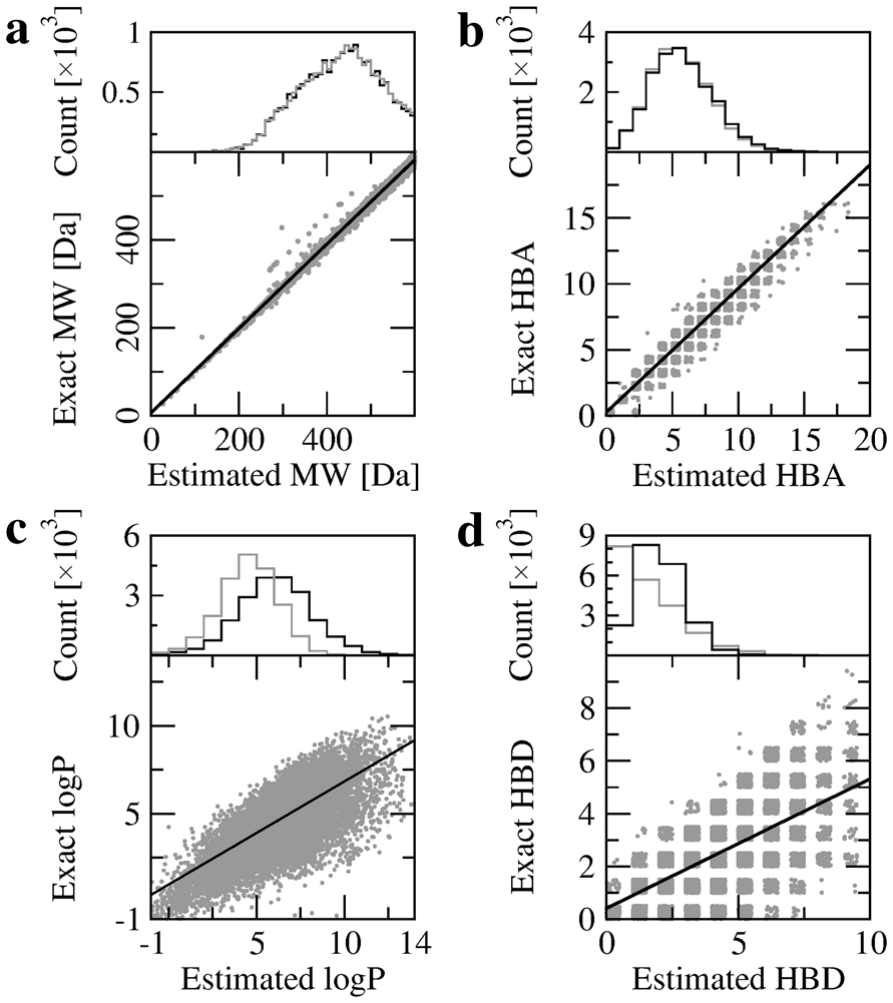
Correlation between exact and estimated Lipinski descriptors. For a given molecule, the following Lipinski’s descriptors are estimated from its fragments: **a** Molecular weight (MW), **b** the number of hydrogen bond acceptors (HBA), **c** the octanol–water partition coefficient (logP), and **d** the number of hydrogen bond donors (HBD). The distribution of the exact (*gray*) and estimated (*black*) properties are shown in histograms attached to correlation plots

The correlation between exact and estimated values of Lipinski’s descriptors are shown in Fig. 5. Figure 5a, b demonstrate a high correlation for correlation for MW and HBA with the Pearson correlation coefficient (PCC) of 0.99 and 0.98, respectively. Figure 5c shows that the PCC between the exact and estimated logP is 0.75. The estimated values tend to be slightly lower than the exact logP, however, the distributions of the estimated and exact values are fairly similar (see the histogram in Fig. 5c). The PCC for HBD is 0.65 (Fig. 5d) with an over-predicted number of hydrogen bond donors. This can be expected since fragments are saturated with hydrogen atoms at positions of covalent bonds in their parent molecules and some of these hydrogens are able to form hydrogen bonds. In order to apply the modified Rule-of-Five, fragment-based physicochemical descriptors are linearly transformed using the regression analysis presented in Fig. 5.

**Fig. 6:**
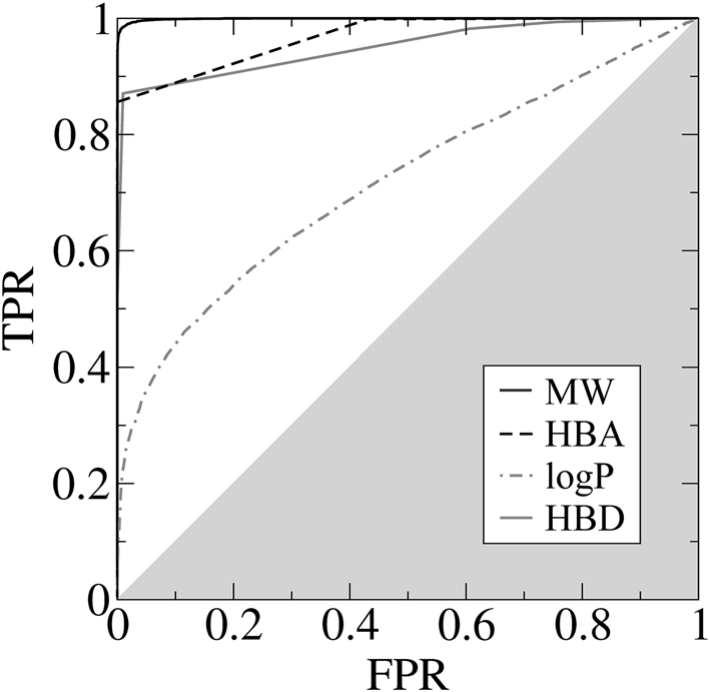
Predicting drug-likeness for molecular synthesis. Receiver operating characteristic plot assessing the accuracy of the prediction of drug-likeness from molecular fragments. The following Lipinski’s descriptors are considered: Molecular weight (MW), the number of hydrogen bond acceptors (HBA), the octanol–water partition coefficient (logP), and the number of hydrogen bond donors (HBD). *TPR* is the true positive rate, *FPR* is the false positive rate, the *gray area* corresponds to the accuracy of a random classifier

Next, we investigate whether Lipinski’s descriptors estimated from molecular fragments can be used to effectively eliminate non-compliant molecules from the synthesis process. Figure 6 presents the receiver operating characteristic (ROC) analysis of a binary classifier based on the MW, HBA, logP and HBD estimates. Here, the accuracy is evaluated by the area under the ROC curve (AUC) calculated for each property. AUC ranges from 0.0 to 1.0, where 1.0 corresponds to the highest accuracy and 0.5 is the accuracy of a random classifier. Encouragingly, MW can be estimated with the highest AUC of 0.999, whereas AUC values for HBA and HBD are 0.967 and 0.948, respectively. Although the correlation between the estimated and exact logP is fairly high (Fig. 5c), the AUC is only 0.717, thus it cannot be used as a reliable predictor of the drug-likeness. This poor discriminatory power is mainly due to the fact that logP values can be either positive or negative. For instance, during molecular synthesis, an intermediate non-polar molecule with logP > 5 can be brought back to the logP below 5 by attaching a highly hydrophilic moiety. Therefore, we only use logP for the final filtering after molecules are synthesized.

### Self-benchmarking test

**Fig. 7:**
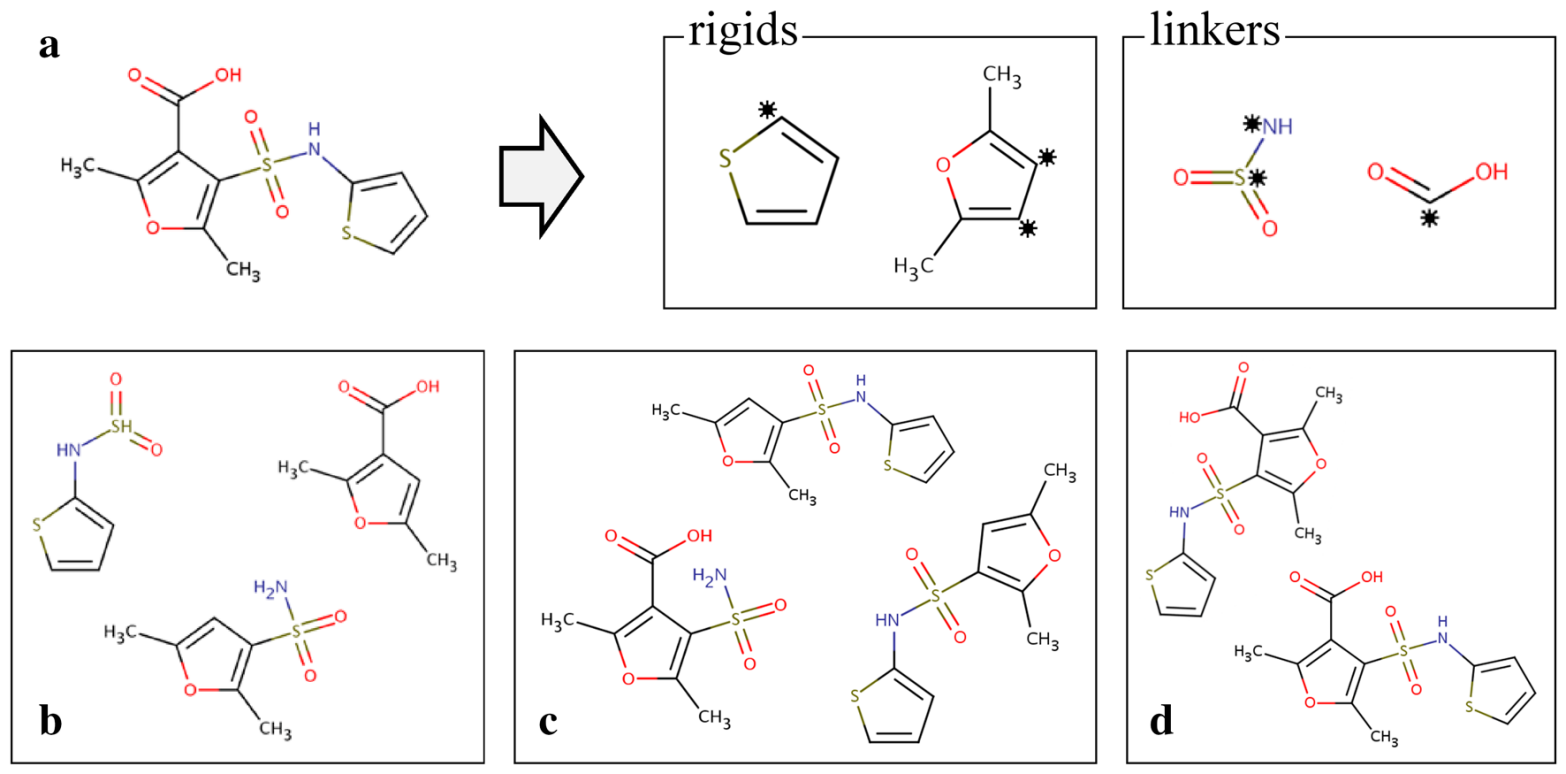
Self-benchmarking example. An example of the successful reconstruction of a molecule from its fragments. **a** The parent molecule is first decomposed into two rigids, thiophene (C_4_H_4_S) and 2,5-dimethylfuran [(CH_3_)_2_C_4_H_2_O], and two linkers, sulfonamide (SO_2_N) and carboxylic acid [C(O)OH]. Examples of constructed **b** *2*-molecules, **c** *3*-molecules, and **d** *4*-molecules including the parent compound

We validate our search algorithm using a self-benchmarking test in which active molecules are reconstructed from their fragments. An example of a successful case is shown in Fig. 7, where the parent compound is decomposed into two rigid and two linker fragments (Fig. 7a). Connecting these fragments through the locations marked by asterisks following the connectivity patterns of the parent molecule produces a series of *2*-, *3*- and *4*-molecules shown in Fig. 7b–d, respectively. The target molecule is correctly reconstructed at level 4 (Fig. 7d).

**Fig. 8:**
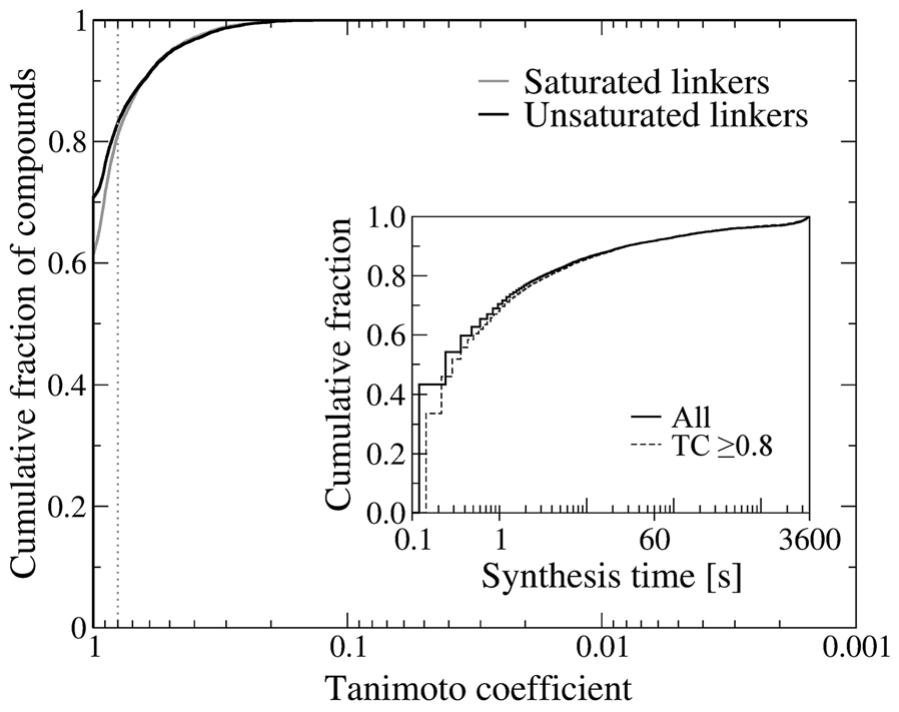
Performance of *e*Synth in the self-benchmarking test. Cumulative fraction of compounds reconstructed with the Tanimoto coefficient (TC) shown on the *x*-axis (logarithmic scale). Saturated linkers (*gray line*) can form all possible connections with rigid fragments, whereas unsaturated linkers (*black line*) can only form as many connections as present in their parent molecules. The *inset* shows the cumulative fraction of all compounds (*solid line*) and successful cases with a TC of ≥0.8 (*dashed line*) computationally synthesized by *e*Synth in 1 h

In Fig. 8, we assess the results obtained for the entire set of 20,408 active compounds from the DUD-E dataset using the highest TC between the synthesized and parent molecules. Using saturated linkers, 61.6 % of actives are reconstructed at a TC of 1.0, whereas 83.1 % have a TC of ≥ 0.8. Moreover, the fraction of actives generated by *e*Synth that match parent compounds increases to 70.9 % when unsaturated linkers are used. Note that Open Babel calculates TC for a pair of ligands using their hashed fingerprints, therefore, a TC of 1.0 denotes identical fingerprints, but not necessarily identical chemical structures. The inset in Fig. 8 shows the computational efficiency of *e*Synth. Here, over 60 % of actives are reconstructed in less than a second using a single processor thread, whereas 90 % compounds are generated within a minute. Note that the synthesis time is fairly similar when only successful cases at a TC of ≥0.8 are considered.

**Fig. 9:**
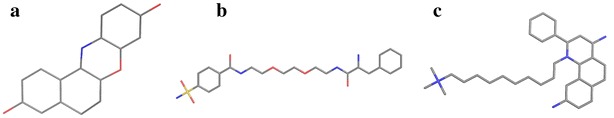
Examples of molecules not reconstructed by *e*Synth. Unsuccessful cases in the self-benchmarking test: **a** A molecule composed of only one rigid fragment, **b**, **c** examples of molecules containing long linkers that exponentially increase the search space

Despite these encouraging results, *e*Synth fails to reconstruct certain molecules. To clarify why some compounds are not correctly generated, Fig. 8 presents main scenarios leading to unsatisfactory results. The first example shown in Fig. 9a is a bioactive compound made up of a single fragment that cannot be decomposed into smaller parts. Since the molecular synthesis is not executed, *e*Synth generates no output. Molecules shown in Fig. 9b, c contain a long linker with a high degree of connectivity. In such cases, rigid fragments can potentially connect to multiple linker locations leading to a combinatorial explosion. In principle, the parent molecule will be reconstructed at some point, however, we limit the wall time for molecular synthesis to 1 h by default. During that time, about 10 % of actives will not be reconstructed as previously shown in Fig. 8 (inset). Using unsaturated linkers, whose connectivity is limited to the original connections in the parent compounds, helps address this issue, nevertheless, those targets containing long and highly flexible linkers are still not generated in a reasonably short computing time. Finally, some compounds are actually correctly reconstructed, yet they are not recognized as similar to their parent molecules. This is a false negative in the assessment of chemical similarity performed by Open Babel; we find that the fingerprint-based matching by Open Babel occasionally fails to recognize the high chemical similarity. We investigate this issue further in the following section.

### Cross-validation test

**Fig. 10:**
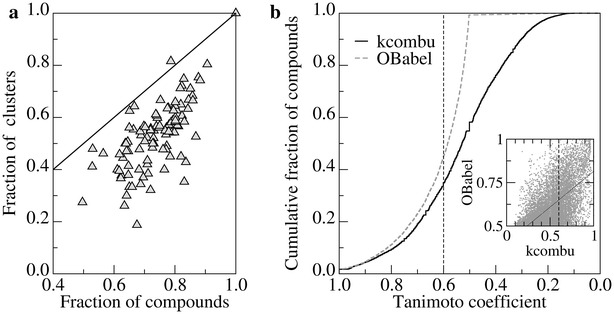
Performance of *e*Synth in the cross-validation test. **a** DUD-E targets depicted as *gray triangles* are positioned in the plot according to the fraction of reconstructible active compounds and the fraction of chemically similar clusters containing only reconstructible actives. Non-reconstructible actives are more uniformly distributed across clusters for those targets lying closer to the *solid black diagonal line*. **b** Cumulative fraction of compounds reconstructed with the Tanimoto coefficient (TC) shown on the *x*-axis. TC is calculated using Open Babel (*dashed gray line*) and kcombu (*solid black line*). The *vertical dashed line* delineates a TC threshold of 0.6. The *inset* shows a direct comparison between TC values computed by Open Babel (1D-TC) and kcombu (2D-TC) with a *solid black regression line*

A cross-validation test was performed in order to evaluate the capability of *e*Synth to generate novel bioactive molecules. Here, we attempt to reconstruct molecules highly similar to target compounds using fragments extracted from chemically different molecules. A set of fragments obtained from clusters other than the target cluster may lack rigid fragment(s) necessary to rebuild some of the active compounds. Since the molecular synthesis algorithm builds on the provided set of fragments, reconstructing molecules without all necessary parts is impossible. Encouragingly, 76.1 % of 23,964 active DUD-E compounds for 101 target proteins are, in principle, reconstructible. Moreover, we examined individual clusters of similar ligands and found out that out of 9406 clusters, as many as 4100 clusters (43.6 %) contain at least one compound that is non-reconstructible because of missing rigid fragments. These numbers are likely underestimated, considering the fact that linker fragments can also be missing and the connectivity patterns may not allow for the correct reconstruction of the topology of target actives, leading to non-reconstructible cases. Interestingly, Fig. 10a indicates that for the majority of DUD-E targets, non-reconstructible actives are typically distributed across clusters of similar molecules.

Figure 10b presents the results obtained for 9406 chemically distinct groups of compounds compiled using active DUD-E ligands. Encouragingly, in 45.1 % (99.3 %) of the cases, the active ligand is reconstructed at a TC of ≥0.6 (≥0.5) using fragments extracted from different clusters associated with the same receptor protein. Here, we employ an ultra-fast implementation of hashed fingerprint-based chemical similarity using Open Babel to compute 1D-TC because of a large number of pairwise similarity calculations. Subsequently, a relatively small fraction of compound pairs, whose 1D-TC is ≥0.5 are subjected to more accurate comparison using 2D-TC by kcombu. In contrast to fingerprint-based techniques, kcombu detects one-to-one chemical matching between two structures that can be used to assess the similarity of their biological activities. When the similarity is evaluated by kcombu, 34.9 % (58.2 %) are reconstructed at a 2D-TC of ≥0.6 (≥0.5). It has been shown that two ligands whose 2D-TC is ≥0.6 typically have similar binding modes with a root-mean-square deviation (RMSD) below 2.0 Å [39, 40]. Moreover, as depicted in the inset in Fig. 10b, 2D-TC of 0.6 reported by kcombu roughly corresponds to a fingerprint-based 1D-TC of 0.7, which is a widely used threshold for similar bioactivity [41–43].

A direct comparison between 1D-TC from Open Babel and 2D-TC from kcombu is shown as the inset in Fig. 10b. The correlation between 1D- and 2D-TC is 0.61 with a somewhat lower similarity assessed by 2D-TC compared to the fingerprint-based 1D-TC. This observation as well as missing fragments leading to non-reconstructible cases explain the lower fraction of compounds successfully reconstructed by *e*Synth when 2D-TC is used to measure the chemical similarity. Since 2D-TC is calculated only for those pairs having 1D-TC ≥ 0.5, the success rate of molecular synthesis is likely underestimated because there are numerous cases for which 2D-TC is actually higher than 1D-TC. This discrepancy between 1D- and 2D-TC also clarifies why a small fraction of target compounds are not recognized as correctly generated in the self-benchmarking test described in the previous section (false negatives). Nonetheless, the results from the cross-validation test performed against a large dataset of bioactive compounds from the DUD-E dataset clearly demonstrate that *e*Synth is capable of generating novel molecules with the desired bioactivities.

### Assessment of the synthetic accessibility

**Fig. 11:**
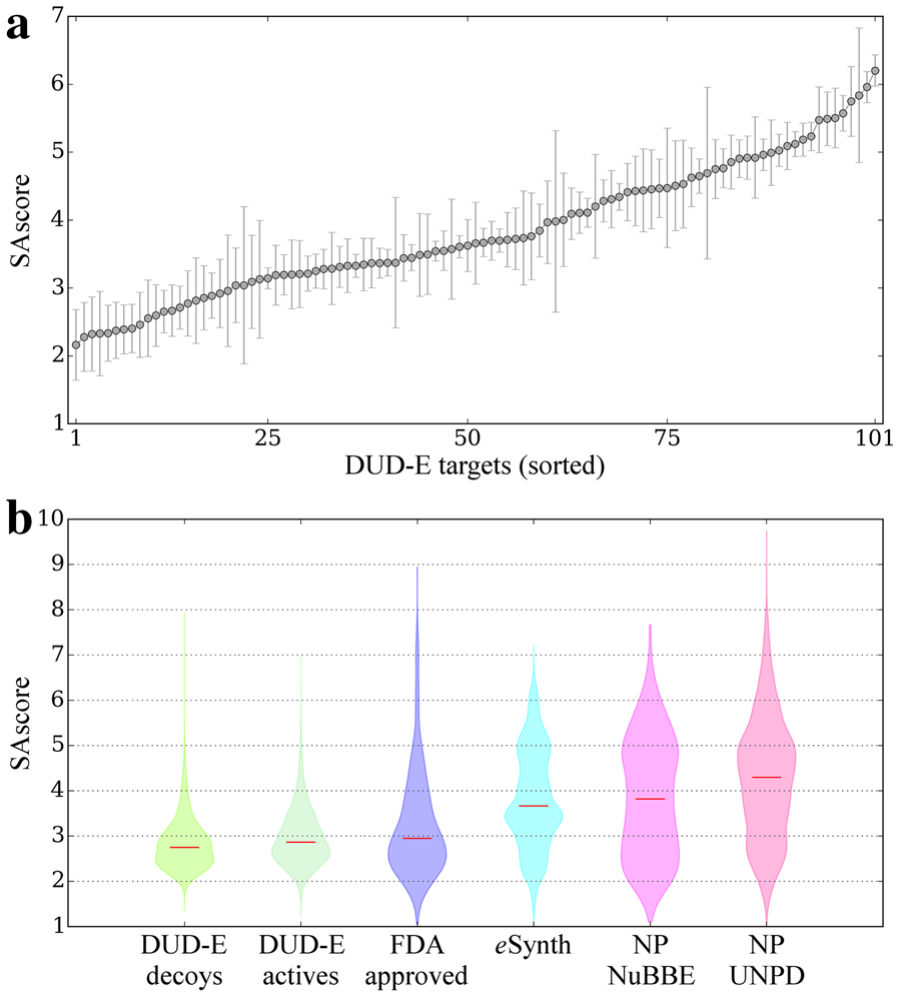
Synthetic accessibility of molecules generated by *e*Synth. **a** Average ± standard deviation synthetic accessibility score (SAscore) calculated for molecules constructed by *e*Synth for individual DUD-E targets (sorted on the *x*-axis). **b** Violin plots showing the distribution of SAscore values across several datasets: decoy and active DUD-E compounds, FDA approved drugs, molecules generated by *e*Synth for the DUD-E targets, and natural products (NP) from the NuBBE and UNPD databases. *Red horizontal lines* correspond to the median SAscore values

Finally, we assess the synthetic accessibility of molecules generated by *e*Synth using SAscore [44]. SAscore employs the synthetic knowledge extracted from already synthesized chemicals penalizing a high molecular complexity; its values range from 1 for easily synthesizable molecules to 10 for those compounds that are very difficult to make. The distribution of SAscore values calculated for molecules generated by *e*Synth in the cross-validation test is shown in Fig. 11. The average SAscore across the DUD-E dataset varies from 2 to 6 (Fig. 11a). In general, molecules with a high SAscore of >6 are difficult to synthesize [44], therefore, the majority of compounds constructed by *e*Synth can be considered as synthetically accessible. Specifically, the average SAscore for compounds generated for 19.8, 61.4, and 87.1 % of DUD-E targets is <3, 4, and 5, respectively. This analysis also demonstrates that the synthetic accessibility depends on a particular biological target and the associated set of bioactive compounds, which are used to extract molecular fragments for *e*Synth.

We also compare the distribution of SAscore values for compounds generated by *e*Synth to those collected for several other datasets. Figure 11b shows that the median SAscore value is 2.75 for decoy and 2.87 for active compounds from the DUD-E dataset (catalogue molecules). Moreover, the median SAscore for FDA approved drugs obtained from DrugBank [45] and compounds constructed by *e*Synth are 2.95 and 3.66, respectively. For comparison, another study reported that the majority of bioactive molecules collected from the Derwent World Drug Index [46] and the MDL Drug Data Report [47] databases have SAscore between 2.5 and 5 [44]. In contrast, natural products are generally more difficult to synthesize than typical organic molecules. Encouragingly, the median SAscore for molecules constructed by *e*Synth is lower than those for natural products, which is 3.82 for the Nuclei of Bioassays, Biosynthesis and Ecophysiology (NuBBE) database of secondary metabolites and derivatives from the biodiversity of Brazil [48], and 4.30 for the Universal Natural Product Database (UNPD) [49]. Compounds from the Dictionary of Natural Products [50] were previously reported to have a broad distribution of SAscore values between 2 and 8. On that account, the synthetic accessibility of molecules generated by *e*Synth is fairly high. The resulting datasets can be further filtered using existing tools, such as SAscore, in order to exclude those compounds containing synthetically unfeasible elements, e.g. chiral centers, large rings and non-standard ring fusions.

## Discussion

Exploring the chemical space to produce pharmacologically applicable compounds is a daunting task because of an enormous size of the search space and numerous biochemical criteria restricting compound generation, i.e. synthetic feasibility, drug-likeness, and the effective binding to the biological target. Using atom-based methods may create an enormous chemical space that can easily surpass the available computing resources. For instance, the largest library generated by an atom-based approach is the GDB-17 dataset comprising 166 billion small molecules [51]. On that account, fragment-based methods can be used as an alternative. Here, reference molecules are used as a source of building blocks, which can be subsequently combined to produce new compounds that are to some extent related to the initial molecules [52]. Fragment-based algorithms typically employ certain rules for combining various moieties, e.g. linker–linker bonds are prohibited, while ring-linker-ring connections are allowed. In contrast to atom-based methods, fragment-based techniques have capabilities to explore much larger molecules.

To facilitate the construction of target-focused libraries for virtual screening, we developed *e*Synth, a new fragment-based approach to molecular synthesis that follows simple combinatorial chemistry steps using an optimized, graph-based algorithm. *e*Synth rapidly generates series of compounds with diverse chemical scaffolds complying with criteria for drug-likeness. Although, these molecules may have different physicochemical properties, the initial fragments are procured from biologically active and synthetically feasible compounds. Consequently, we demonstrated that the constructed libraries are enriched with pharmacologically relevant molecules synthesized under loose biochemical constraints.

Our effort simplifies the synthesis process by avoiding techniques such as click chemistry, e.g. AutoClickChem [53], and those relying on statistical restrains, e.g. Fragment Optimized Growth (FOG) [54]. Moreover, in contrast to other methods designed for certain classes of compounds such as peptides generated from amino acid fragments, e.g. GrowMol [55] and LUDI [56], *e*Synth can construct any class of organic, drug-like molecules. Several methods employ the binding site information in order to generate molecules with a binding affinity toward a given target protein, e.g. Multiple Copy Simultaneous Search (MCSS) [57], SPROUT: structure generating software using template [58], and SMall Molecule Growth (SMoG) [59]. *e*Synth does not require protein structures, yet the cross-validation test clearly demonstrates that molecules highly similar to those compounds known to bind to the target protein are effectively generated.

Evolutionary algorithms that break fragments and make crossovers allow for an exhaustive exploration of the chemical space [60, 61], however, using these techniques also requires applying chemical stability and synthetic feasibility rules, which, in turn, utilizes extra computational resources. For instance, the Algorithm for Chemical Space Exploration with Stochastic Search (ACSESS) was designed to construct representative universal libraries in an arbitrary chemical space [61]. This approach implements convergent evolutionary operations through bond and/or atom modifications on an initial library of molecules to acquire a maximally diverse subset of molecules. Although using evolutionary techniques does not guarantee a completeness of the space search, ACSESS systematically explores the small molecule universe, providing a near-infinite source of novel compounds. Differ from other techniques employing generic combinatorial algorithms, chemical rules and filters, *e*Synth was not designed to explore a broad chemical space; rather, it is purposely confined to a chemical sub-space around a particular drug target.

*e*Synth relies solely on fragments and their connectivity patterns extracted from parent molecules to generate a series of drug-like compounds. Thus, it is essential to use synthetically feasible bioactive compounds as the source in order to generate molecules with similar chemical and pharmacological profiles. Importantly, *e*Synth is not restricted to a particular hypothesis, e.g. a pre-defined pharmacophore often used by synthesis algorithms. For example, a pharmacophore-based *de novo* design method of drug-like molecules (PhDD) ensures that molecules constructed from linker and rigid fragments fit a given pharmacophore model [60]. The search space in PhDD is not only confined to the fragment and linker libraries, but also it is limited to a user-defined template molecule in the form of a pharmacophore hypothesis. *e*Synth avoids such hypotheses in order to generate target-focused compound datasets, yet without any bias toward a specific scaffold.

Molecular synthesis methods often use knowledge-based rules to connect fragments. For example, combining the amine with the carbonyl to form the amide changes the preference of the nitrogen atom toward those moieties that might be more likely attached to an amide rather than an amine nitrogen [54]. On that account, FOG uses the statistical knowledge to create new branches and decide which branch to grow as an effective way to generate novel molecules. Similar to *e*Synth, FOG employs a construction algorithm using molecular fragments to generate synthetically tractable molecules, however, it grows molecules using a Markov Chain according to statistics on the frequency of specific connections in the database of chemicals. Moreover, the Topology Classifier algorithm is used to classify the constructed molecules as drugs or non-drugs. Given a set of fragments, the chemical search space in FOG may be somewhat limited to those molecules having similar characteristics as the training compounds. In contrast, *e*Synth creates new molecules by reusing fragments and following their connectivity patterns in the parent compounds. Therefore, it the covers a larger chemical space and does not require constructing statistical databases of fragment connections.

## Conclusions

*e*Synth is a new algorithm to generate large datasets of chemical compounds by connecting small molecular fragments. It first establishes the width of a search space with a diverse foundation of initial small molecules followed by the stochastic exploration of the depth of the chemical space by constructing multi-fragment molecules. This hybrid approach ensures a deeper exploration of the molecular space by synthesizing larger molecules while circumventing the necessity of a complete exploration through the synthesis of all possible molecules. *e*Synth can compile large libraries of drug-like molecules with the desired properties, which may be unfeasible using atom-based synthesis techniques. Moreover, the resulting libraries can be further filtered based on the geometry and energy of binding, and the biological activity toward specific targets. Finally, we demonstrated that *e*Synth has capabilities to generate novel, biologically active ligands for target proteins from chemically distinct parent molecules.

## Availability and requirements

- Project name: *e*Synth
- Project home page: www.brylinski.org/content/molecular-synthesis
- Operating system(s): Platform independent, preferably Linux
- Programming language: C ++
- Other requirements: Open Babel, GSL, Zlib
- License: GNU GPL
- Any restrictions to use by non-academics: license needed
